# Oxidation States Regulation of Cobalt Active Sites through Crystal Surface Engineering for Enhanced Polysulfide Conversion in Lithium–Sulfur Batteries

**DOI:** 10.1002/advs.202202352

**Published:** 2022-09-15

**Authors:** Rujian Xiao, Dan Luo, Jiayi Wang, Han Lu, Heng Ma, Eser Metin Akinoglu, Mingliang Jin, Xin Wang, Yongguang Zhang, Zhongwei Chen

**Affiliations:** ^1^ South China Academy of Advanced Optoelectronics School of Information and Optoelectronic Science and Engineering South China Normal University Guangdong 510006 China; ^2^ Department of Chemical Engineering University of Waterloo Waterloo ON N2L 3G1 Canada; ^3^ International Academy of Optoelectronics at Zhaoqing South China Normal University Zhaoqing 526060 China; ^4^ School of Materials Science and Engineering Hebei University of Technology Tianjin 300130 China

**Keywords:** crystal surface engineering, lithium–sulfur batteries, oxidation states, polysulfide conversion

## Abstract

In this work, unique Co_3_O_4_/N‐doped reduced graphene oxide (Co_3_O_4_/N‐rGO) composites as favorable sulfur immobilizers and promoters for lithium–sulfur (Li–S) batteries are developed. The prepared Co_3_O_4_ nanopolyhedrons (Co_3_O_4_‐NP) and Co_3_O_4_ nanocubes mainly expose (112) and (001) surfaces, respectively, with different atomic configurations of Co^2+^/Co^3+^ sites. Experiments and theoretical calculations confirm that the octahedral coordination Co^3+^ (Co^3+^_Oh_) sites with different oxidation states from tetrahedral coordination Co^2+^ sites optimize the adsorption and catalytic conversion of lithium polysulfides. Specially, the Co_3_O_4_‐NP crystals loaded on N‐rGO expose (112) planes with ample Co^3+^_Oh_ active sites, exhibiting stronger adsorbability and superior catalytic activity for polysulfides, thus inhibiting the shuttle effect. Therefore, the S@Co_3_O_4_‐NP/N‐rGO cathodes deliver excellent electrochemical properties, for example, stable cyclability at 1 C with a low capacity decay rate of 0.058% over 500 cycles, superb rate capability up to 3 C, and high areal capacity of 4.1 mAh cm^−2^. This catalyst's design incorporating crystal surface engineering and oxidation state regulation strategies also provides new approaches for addressing the complicated issues of Li–S batteries.

## Graphical abstract

Synthesis of Co_3_O_4_ nanopolyhedron/N‐doped reduced graphene oxide (Co_3_O_4_‐NP/N‐rGO) host materials by crystal surface engineering and oxidation state regulation strategies for high‐performance lithium–sulfur batteries is reported. The as‐prepared Co_3_O_4_‐NP/N‐rGO composites mainly expose (112) surfaces with ample octahedral coordination Co^3+^ active sites exhibiting strong adsorption of polysulfides and enhanced redox reaction kinetics, thus resulting in excellent cycling stability and rate capability of batteries.

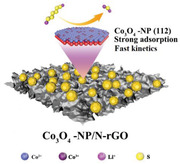

## 1 Introduction

Lithium–sulfur (Li–S) batteries hold great promise as the energy storage systems due to their theoretical specific energy density (2600 Wh kg^−1^), which is much higher than that of conventional lithium‐ion batteries.^[^ ^1^, ^2^, ^3^, ^4^ ^]^ However, the practical application of the Li–S batteries has been hindered by several technical issues: the insulating character of sulfur (S_8_) and lithium sulfide (Li_2_S), the huge volume change of S_8_ during charge–discharge process, the notorious lithium polysulfides (LiPSs) shuttle effect, and sluggish sulfur conversion kinetics, which lead to low specific capacity, poor rate capability, and cycle performance.^[^ ^5^, ^6^, ^7^, ^8^ ^]^ In order to solve these problems, various nanostructured carbon materials, such as carbon fibers, graphene oxide (GO), and carbon nanotubes, have been widely used as conductive host materials for sulfur and to confine polysulfides.^[^ ^9^, ^10^, ^11^, ^12^, ^13^ ^]^ Although nonpolar carbon materials can provide a large electrode–electrolyte contact area, cushion volume expansion of sulfur, and shorten diffusion paths of electron and ion transport, they cannot efficiently anchor polar polysulfides, resulting in serious capacity degradation.^[^ ^14^, ^15^, ^16^, ^17^ ^]^ It is reported that nitrogen‐doping can facilitate electron conductivity and electrochemical reaction, and effectively improve the surface properties of carbon materials.^[^ ^18^, ^19^, ^20^ ^]^ In particular, nitrogen‐doping will greatly affect the intrinsic physicochemical properties of graphene, such as lithium storage capacity and conductivity, and inhibit the LiPSs shuttling through coordinating with N atoms.^[^ ^21^, ^22^, ^23^, ^24^ ^]^

Given that LiPSs are polar substances with negative charge, polar host materials have been gradually developed. Compared with carbon materials, the metal oxides with ample polar sites can strongly bind to LiPSs through Lewis acid–base interaction, polar–polar interaction, and sulfur–chain catenation, so as to adsorb them on the host surface and reduce the loss of active substances.^[^ ^25^ ^]^ Metal oxides, such as Co_3_O_4_, MoO_2_, and TiO_2_, have been proved to be effective in improving the stability of Li–S batteries.^[^ ^26^, ^27^, ^28^, ^29^ ^]^ Moreover, the shuttle effect can also be improved by electrocatalysis. The introduction of catalytic metals (e.g., Co, Pt, and Ni) will significantly accelerate the conversion of high‐order LiPSs.^[^ ^30^, ^31^, ^32^ ^]^ What's more interesting is that the Co‐based particles can greatly enhance the electrochemical performance of batteries.^[^ ^33^ ^]^ The spinel type Co_3_O_4_ with a Co^2+^Co^3+^_2_O_4_ structure, in which the Co^3+^ and Co^2+^ ions occupy the octahedral and tetrahedral sites, respectively, is recognized as an excellent electrocatalyst.^[^ ^34^ ^]^ It is well known that the performance of catalyst is closely related to its electronic structure. The catalytic performance of the Co_3_O_4_ can be remarkably changed when tuning its electronic structure.^[^ ^35^ ^]^

The surface atomic configurations vary in different lattice planes of Co_3_O_4_ nanocrystals, leading to differences in the surface atomic density, surface electronic structures, geometric bonding, and chemical reactivity,^[^ ^36^, ^37^ ^]^ which have a significant impact on physicochemical properties and electrochemical performances of crystal surfaces.^[^ ^38^, ^39^, ^40^ ^]^ It is worth noting that through crystal surface engineering, exposing different crystal faces and adjusting the catalytic sites on the Co_3_O_4_ surface can enable appropriate bond strength between substrates and active sites, thus improving the catalytic performance.^[^ ^38^, ^41^, ^42^ ^]^ From the atomic arrangements of Co_3_O_4_ (001) and Co_3_O_4_ (112) surfaces (Figure S1, Supporting Information), the (001) surface contains only tetrahedral coordination Co^2+^(Co^2+^_Td_) sites, while the (112) surface contains both Co^2+^_Td_ and octahedral coordination Co^3+^(Co^3+^_Oh_) sites. It can be predicted that the Co_3_O_4_ (001) and Co_3_O_4_ (112) will exhibit different catalytic behaviors due to the regulation of oxidation states, which is able to enhance the LiPSs catalytic conversion toward amplified Li–S electrochemistry.

Herein, we here designed the Co_3_O_4_ nanopolyhedron (Co_3_O_4_‐NP) and Co_3_O_4_ nanocube (Co_3_O_4_‐NC) with different dominant lattice planes on N‐doping reduced graphene oxide (N‐rGO) by a simple hydrothermal method as sulfur electrocatalysts in Li–S batteries. Experimental results and theoretical calculations show that the Co^3+^_Oh_ species have stronger coupling and catalytic effect on LiPSs than Co^2+^_Td_ species, which is attributed to the different oxidation states of the two components. Accordingly, compared with Co_3_O_4_‐NC (001), Co_3_O_4_‐NP (112) crystal planes, which consist abundant Co^3+^_Oh_ cations, can not only provide potent sulfur immobilization to effectively inhibit shuttle effect, but also dynamically accelerate LiPSs catalytic conversion to realize superior sulfur redox kinetics, giving rise to amplified Li–S performance.

## 2 Results and Discussion

**Figure 1:**
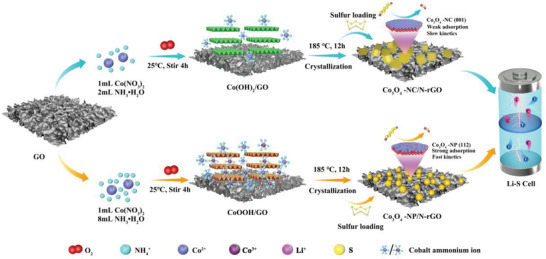
Schematic illustration of Co_3_O_4_‐NC/N‐rGO and Co_3_O_4_‐NP/N‐rGO composites prepared by hydrothermal method.

The preparation process and formation mechanism of S@Co_3_O_4_/N‐rGO composites are schematically illustrated in **Figure** **1**. By simply adjusting the amount of ammonia, the Co_3_O_4_ crystals supported on GO can transform from nanocube to nanopolyhedron. At a high concentration of NH_3_·H_2_O, the Co(NH_3_)_6_^2+^ in the solution will be oxidized by oxygen to Co(NH_3_)_6_^3+^, forming a stable colloidal CoOOH solution rather than layered Co(OH)_2_.^[^ ^43^, ^44^ ^]^ In comparison with green Co(OH)_2_, the presence of Co^3+^ ions in brown CoOOH intermediates is proposed to explain the formation of the (112) plane oriented Co_3_O_4_‐NP crystals. Meanwhile, in the hydrothermal reaction, the ammonia acts as a nitrogen source to convert GO to N‐rGO. The N‐rGO can offer sufficient growing sites for metal oxide nuclei to generate Co_3_O_4_/N‐rGO composites.^[^ ^45^ ^]^

**Figure 2:**
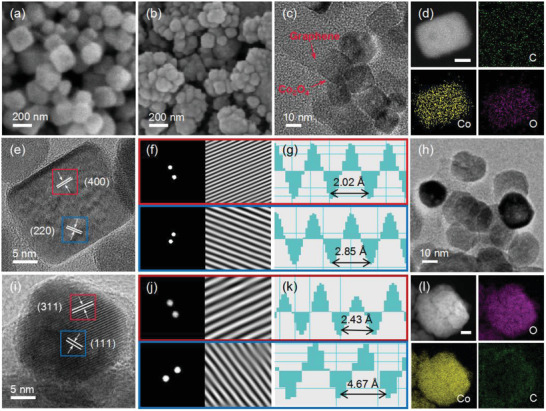
a,b) SEM, c,h) TEM, and e,i) HRTEM images of Co_3_O_4_‐NC/N‐rGO and Co_3_O_4_‐NP/N‐rGO. f,j) Inverse FFT patterns of selected areas. g,k) Corresponding lattice spacing profiles. d,l) Elemental mapping of Co_3_O_4_‐NC/N‐rGO and Co_3_O_4_‐NP/N‐rGO with a scale bar of 5 and 20 nm.

As shown in **Figure** **2**, the morphology and crystal structure of the obtained Co_3_O_4_ crystals were investigated by electron microscopy. The clear lattice fringes observed in high‐resolution transmission electron microscopy (HRTEM) and inverse fast Fourier transformation (FFT) patterns reveal the crystallographic nature of the spinel structure. The Co_3_O_4_‐NC crystals are monodispersed on the rGO with a uniform size of 20 nm which also demonstrates perfect edges and clear surfaces. The lattice resolved HRTEM image shows the (220) and (400) crystal planes with 2.85 and 2.02 Å *d*‐spacings. When the observed interface angle is 45°, it indicates that the projected direction is (001), which confirms that the dominant exposed surfaces of Co_3_O_4_‐NC crystals are (001) planes (Figure 2e–g and Figures S2 and S3, Supporting Information).^[^ ^46^, ^47^ ^]^ The main exposed surface of Co_3_O_4_‐NP crystals with an average size of about 18 nm is the (112) surface. We can observe the (311) and (111) crystal planes along the (112) zone axis, which have 2.43 and 4.67 Å lattice spacing, respectively (Figure 2i–k and Figures S4 and S5, Supporting Information).^[^ ^48^ ^]^ In addition, the energy‐dispersive X‐ray spectra of Co_3_O_4_/N‐rGO composites are depicted in Figure 2d,l, which show the uniformed distribution of C, Co, and O elements. Scanning electron microscopy (SEM) images also confirmed the morphologies of these two kinds of nanomaterials (Figure 2a,b).

**Figure 3:**
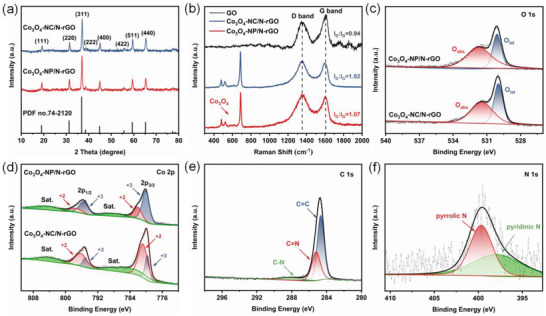
a) XRD patterns. b) Raman spectra. c) O 1s and d) Co 2p high‐resolution XPS spectra. e) C 1s and f) N 1s of Co_3_O_4_‐NP/N‐rGO.

The phase and composition of the products were characterized by X‐ray diffraction (XRD) patterns and Raman spectra. Despite variations in shape, the identified peaks are indexed as the fcc spinel type Co_3_O_4_ (JCPDS No. 74‐2120) and the patterns display high crystallinity and purity of the Co_3_O_4_ crystals (**Figure** **3**a). In addition, some diffraction peaks of S@Co_3_O_4_/N‐rGO composites are basically consistent with orthonormal sulfur (JCPDS No. 08‐0247) (Figure S6, Supporting Information).^[^ ^49^ ^]^ The SEM images and element mapping presented in Figure S7, Supporting Information, further demonstrate the uniform sulfur distribution. As revealed by Raman results (Figure 3b), two peaks at 1590 and 1350 cm^−1^ can be attributed to the G bands (graphitic sp^2^ stretching) and D bands (disordered sp^3^) of graphene, respectively.^[^ ^50^ ^]^ In the range from 400 to 800 cm^−1^, three distinct bands at 478 (E_2g_), 521 (F_2g_), and 686 cm^−1^ (A_1g_) can be observed, corresponding to the feature of spinel Co_3_O_4_.^[^ ^51^ ^]^ According to the intensity ratios of D‐band to G‐band(*I*_D_/*I*_G_) of GO, Co_3_O_4_‐NC/N‐rGO, and Co_3_O_4_‐NP/N‐rGO (0.94, 1.02, and 1.07), the graphite structure in rGO becomes more disordered obviously. Furthermore, three sharp peaks appearing below 500 cm^−1^ in the Raman spectrum can be ascribed to elemental sulfur, confirming the formation of the S@Co_3_O_4_/N‐rGO composites (Figure S8, Supporting Information).

To examine the surface composition and chemical bonding of Co_3_O_4_/N‐rGO composites, X‐ray photoelectron spectroscopy (XPS) characterization was performed (Figure 3c–f and Figure S9, Supporting Information). The high‐resolution XPS spectra of N 1s show two peaks related to the pyrrolic (399.7 eV) and pyridinic (397.9 eV) nitrogen, which proves that nitrogen atoms are successfully doped into graphene.^[^ ^52^, ^53^, ^54^ ^]^ According to Table S1, Supporting Information, the XPS survey spectra of Co_3_O_4_‐NC/N‐rGO and Co_3_O_4_‐NP/N‐rGO show similar elemental compositions of C, Co, O, and N. The atomic ratio of nitrogen/carbon (N/C) is about 2.1%, indicating that the N doping rates of the two composites are the same. There are two peaks located at 795.5 and 780.2 eV in the Co 2p spectra, corresponding to the Co 2p_1/2_ and Co 2p_3/2_ orbits. The energy difference between Co 2p_1/2_ and Co 2p_3/2_ splitting is about 15 eV, indicating the existence of low spin Co^2+^.^[^ ^55^, ^56^ ^]^ Besides, the high resolution of Co 2p_3/2_ in Co_3_O_4_‐NP/N‐rGO can be deconvoluted into two peaks at 782.1 and 780.1 eV, which can be ascribed to Co^2+^ and Co^3+^ species.^[^ ^47^, ^57^ ^]^ The peaks at 797.5 and 795.5 eV can be attributed to the spin–orbital properties of Co 2p_1/2_. Obviously, the Co_3_O_4_‐NP/N‐rGO has a higher relative area ratio of Co^3+^ to Co^2+^ (Figure S10, Supporting Information), implying the Co_3_O_4_‐NP (112) surface with more Co^3+^ ions. As shown in Figure 3c, two peaks located at 531.7 and 530.1 eV in the O 1s spectra can be assigned to adsorbed oxygen (O_ads_) and crystal lattice oxygen (O_lat_) of Co_3_O_4_, individually.^[^ ^58^ ^]^ The C 1s peaks at binding energies of 287.5, 285.2, and 284.7 eV derive from contributions of different C—N, C=N, and C=C type species of N‐doped graphene (Figure 3e).^[^ ^59^ ^]^ In addition, the Co_3_O_4_‐NC/N‐rGO and Co_3_O_4_‐NP/N‐rGO materials exhibit similar specific surface area, which enables us to exclude the effect of specific surface area on the performance and pay more attention to the role of Co active sites in the electrocatalytic behaviors (Figure S11, Supporting Information).

**Figure 4:**
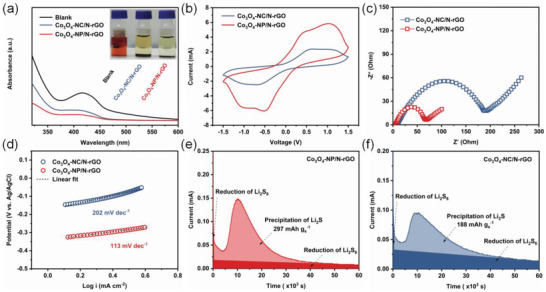
a) UV–vis spectra of adsorption test (inset image: optical photographs of Li_2_S_6_ solution after adsorption by various materials). b) CV curves at 10 mV s^−1^ and c) EIS spectra of symmetric cells. d) Tafel plots of Li_2_S oxidization on Co_3_O_4_‐NC/N‐rGO and Co_3_O_4_‐NP/N‐rGO surfaces. e,f) Li_2_S deposition profiles.

To verify that the Co_3_O_4_‐NP/N‐rGO composites have stronger adsorption ability toward LiPSs, the same amount of two host materials were added into Li_2_S_6_ solutions separately. Clearly, after adsorption by Co_3_O_4_‐NP/N‐rGO, the color of Li_2_S_6_ solution gradually faded, while the color of the one with Co_3_O_4_‐NC/N‐rGO slowly changed to light yellow. The UV–vis spectroscopy was also used to analyze the concentration changing of Li_2_S*_x_* (*x* = 4–8) solution. The intensity of absorption peak located at about 425 nm in the spectra can indicate the amount of S_4_^2−^ species.^[^ ^60^ ^]^ The solution with Co_3_O_4_‐NP/N‐rGO delivers a much lower absorbance peak intensities than those containing Co_3_O_4_‐NC/N‐rGO or blank (Li_2_S_6_ solution), confirming its stronger LiPSs adsorbability (**Figure** **4**a). As a result, the effective exposure of the Co^3+^ active sites is beneficial to LiPSs adsorption, which provides a better chemical constraint for LiPSs shuttle inhibition. In addition to LiPSs adsorbability, the catalytic effect also plays an important role in improving the electrochemical performance of sulfur cathode. Symmetrical cells using identical Co_3_O_4_/N‐rGO as electrodes were assembled with the electrolyte containing Li_2_S_6_. The cyclic voltammetry (CV) profile apparently exhibits larger and sharper redox peaks with the smaller polarizations for Co_3_O_4_‐NP/N‐rGO, suggesting the more sufficient LiPSs transformation and faster reaction kinetics (Figure 4b). Different scan rate CV curves and the linear relations between peak current (*I*_p_) and square root of scanning rate (*v* ^0.5^) demonstrate that the Li^+^ diffusion coefficient (*D*_Li_^+^) of Co_3_O_4_‐NP/N‐rGO electrode is higher than that of Co_3_O_4_‐NC/N‐rGO (Figure S12, Supporting Information). Moreover, the electrochemical impedance spectroscopy (EIS) was carried out, which shows a smaller charge transfer resistance for the Co_3_O_4_‐NP/N‐rGO cathode, proving the facile sulfur reactions and its fast charge/mass‐transfer process (Figure 4c).

Apart from that, the oxidation behaviors of Li_2_S on different catalytic planes and catalytic activity of Co_3_O_4_/N‐rGO composites were investigated by linear sweep voltammetry in a three‐electrode configuration (Figure 4d and Figure S13, Supporting Information). The Co_3_O_4_‐NP/N‐rGO electrode delivers a lower onset potential of −0.36 V and smaller Tafel slope of 113 mV dec^−1^ in comparison to Co_3_O_4_‐NC/N‐rGO (−0.21 V, 202 mV dec^−1^), evidencing its lower overpotential for Li_2_S oxidation and the kinetic enhancement. The redox kinetics of sulfur also can be reflected by the deposition behavior of Li_2_S.^[^ ^61^, ^62^ ^]^ Remarkably, the cell based on Co_3_O_4_‐NP/N‐rGO exhibits higher precipitation capacity (297 mAh g^−1^) compared with the Co_3_O_4_‐NC/N‐rGO (188 mAh g^−1^) and stronger current response, indicating the lower energy barriers for the nucleation and growth of Li_2_S on Co_3_O_4_‐NP/N‐rGO surface (Figure 4e,f).

Previous studies have proved that the catalytic activity of metal oxide nanoparticles came from ion pairs (catalytic sites in two different oxidation states).^[^ ^63^ ^]^ The surface layer of Co_3_O_4_ (001) plane contains Co^2+^ ions, without any Co^3+^ ions, but there are two Co^3+^ ions in the sub‐layer which are usually active sites for catalysis.^[^ ^64^ ^]^ However, it is difficult for substrate to contact with the Co^3+^ ions of Co_3_O_4_ nanocube in a catalytic reaction. The Co_3_O_4_ nanopolyhedron predominantly exposes the (112) surface with ample Co^3+^ active sites, promoting the chemical interactions between Co_3_O_4_ and LiPSs. At the same time, the Co^3+^ cations act as catalytic sites to expedite the LiPSs conversion and contribute significantly to reaction. Consequently, the Co_3_O_4_‐NP enclosed by (112) plane exhibits higher catalytic activity than the Co_3_O_4_‐NC with (001) plane.

**Figure 5:**
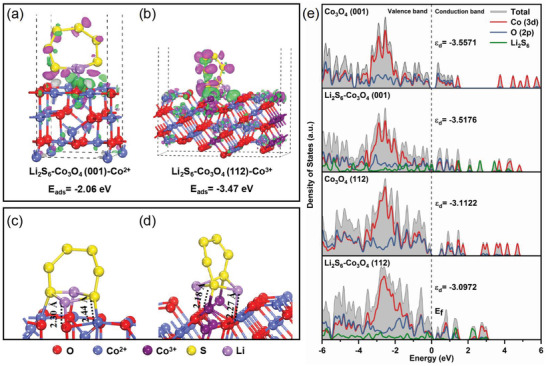
Charge density difference for the geometrical configurations of Li_2_S_6_ molecules interacting with Co sites at different oxidation states: a) Li_2_S_6_‐adsorbed Co_3_O_4_ (001)‐Co^2+^, b) Li_2_S_6_‐adsorbed Co_3_O_4_ (112)‐Co^3+^. The bond length of Co—S and Li—O in stable configurations of Li_2_S_6_ adsorption on c) Co_3_O_4_ (001) and d) Co_3_O_4_ (112) surfaces. e) Total and partial DOS of Co_3_O_4_ (001), Co_3_O_4_ (112) surfaces, Li_2_S_6_‐Co_3_O_4_ (001), and Li_2_S_6_‐Co_3_O_4_ (112).

To gain further insight into the experimental mechanism, density functional theory calculation was carried out with the optimized geometries of Li_2_S_6_ adsorption on ideal Co_3_O_4_ (001) and Co_3_O_4_ (112) surfaces. According to the charge accumulate (red) and dissipate (green) position, the electrons tend to transport from Co and Li to S and O atoms (**Figure** **5**a,b). More charge transfer can be observed in Co_3_O_4_ (112) facet than the Co_3_O_4_ (001) facet, which confirms that the exposure of high‐valent (+3) Co cations can attract more electrons and effectively boost the electron transfer. The adsorption configuration of Li_2_S_6_ on Co_3_O_4_ (001)‐Co^2+^ exhibits chemical interaction by forming Co—S bond with a bond length of 2.44 Å. In stark contrast, shorter bond length of Co—S (2.18 Å) can be found in Li_2_S_6_‐Co_3_O_4_ (112)‐Co^3+^, implying much stronger interaction than that on Co_3_O_4_ (001)‐Co^2+^ (Figure 5c,d and Figure S14, Supporting Information). Evidently, the adsorption energies of Li_2_S_6_ on Co_3_O_4_ (001) and Co_3_O_4_ (112) surfaces are −2.06 and −3.47 eV respectively, suggesting the stronger surface affinity of Co_3_O_4_ (112) for LiPSs and chemical sulfur immobilization. Different coupling strengths between Co‐3d and anion p orbitals in these two geometries can also be seen from the density of states (Figure 5e and Figures S15–S18, Supporting Information). Compared with Co_3_O_4_ (001), the d‐band center of Co 3d in Co_3_O_4_ (112) (−3.1122 eV) has a distinct upshift with respect to Fermi level, revealing the favorable charge transfer and higher charge density during sulfur catalytic processes of the Co_3_O_4_ (112). After adsorption of Li_2_S_6_, the partial density of states (PDOS) of Li_2_S_6_‐Co_3_O_4_ (001) as well as Li_2_S_6_‐Co_3_O_4_ (112) also displays similar characteristics and the Fermi level lies below the valence band edge which means that the conductivity of the Co_3_O_4_ surface is enhanced. The PDOS shows a reduced energy gap between the anion p and Co 3d band centers of Li_2_S_6_‐Co_3_O_4_ (112) compared to Li_2_S_6_‐Co_3_O_4_ (001). This further leads to reduced antibonding orbitals, increased energy of bonding states, higher electron energy, and electrochemical activity, thereby inducing stronger chemisorption with LiPSs and modulating the electron exchange to promote the interfacial S_6_^2−^/S^2−^ redox dynamics. In addition, more peaks appear around the Fermi level mainly due to the orbital contribution of the S atoms. The Co_3_O_4_ (112) achieves a higher electronic concentration at Fermi level after adsorbing Li_2_S_6_, proving that Co_3_O_4_ (112) has better conductivity than Co_3_O_4_ (001). Hence, the oxidation state of Co (+3 or +2) determines the electron distribution of the Co‐3d orbit, which remarkably changes the interaction mode of Li_2_S_6_ toward Co and affects its catalytic properties. The presence of Co^3+^ improves the adsorption energy and strengthens electron exchange with sulfur‐containing intermediates, indicating the enhanced catalytic activity of Co_3_O_4_ (112).

**Figure 6:**
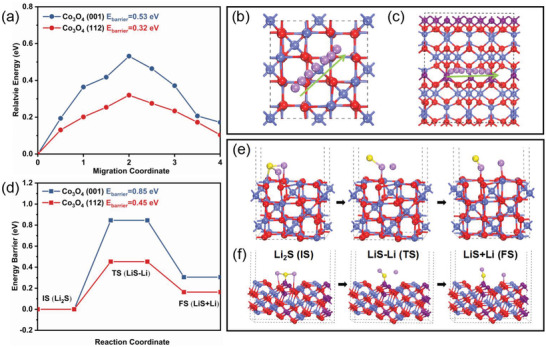
a) Diffusion profiles of Li^+^ on different surfaces. Diffusion paths of Li^+^ on b) Co_3_O_4_ (001) and c) Co_3_O_4_ (112) surfaces. d) Energy profiles of Li_2_S decomposition on Co_3_O_4_ (001) and Co_3_O_4_ (112) surfaces. Geometrical configurations of Li_2_S decomposition on e) Co_3_O_4_ (001) and f) Co_3_O_4_ (112) surfaces.

The diffusion behavior of Li^+^ can also be used to evaluate the potential kinetic improvement by crystal surface engineering. Diffusion diagrams and corresponding diffusion paths of lithium ions on different crystal planes are described in **Figure** **6**a–c. Obviously, the diffusion barrier energy of Li^+^ on Co_3_O_4_ (112) surface is 0.32 eV, which is lower than the 0.53 eV on Co_3_O_4_ (001) surface, indicating that the crystal surface engineering promotes ion transfer. It is well known that random deposition of Li_2_S is the cause of rapid decay of battery capacity.^[^ ^65^ ^]^ To investigate the kinetic process, the climbing‐image nudged elastic band method is applied to calculate the activation energies of Li_2_S decomposition. Figure 6e,f shows the geometric configuration of the initial structure (IS), transition structure (TS), and final structure (FS) for Li_2_S decomposition on Co_3_O_4_ (001) and Co_3_O_4_ (112) surfaces. In the energy profiles, the decomposition energy barrier (0.45 eV) of Li_2_S on Co_3_O_4_ (112) plane is lower than that on Co_3_O_4_ (001) plane (0.85 eV), proving the facilitated Li_2_S transformation kinetics by exposing Co^3+^ active sites (Figure 6d).

**Figure 7:**
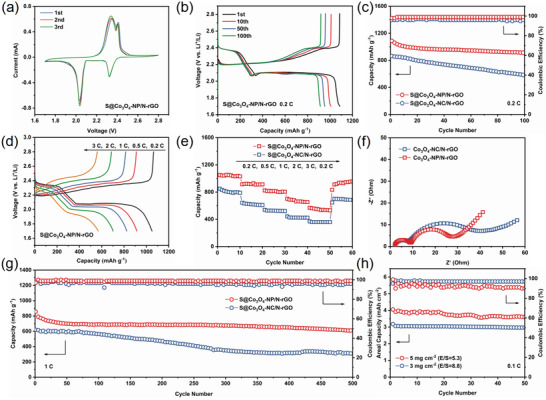
a) CV plots and b) charge–discharge profiles of S@Co_3_O_4_‐NP/N‐rGO. c) Cycling performance at 0.2 C, d,e) rate capability, f) EIS spectra, and g) long‐term cycling stability at 1 C of different electrodes. h) High‐loading cycling behavior of S@Co_3_O_4_‐NP/N‐rGO electrode at 0.1 C.

The electrochemical properties of the obtained S@Co_3_O_4_‐NP/N‐rGO electrodes were investigated. According to the TGA curves (Figure S19, Supporting Information), the weight loss of about 75% from 30 to 300 °C is due to the sulfur, the weight loss of ≈7% between 300 and 750 °C owes to the oxidation of carbon, and the remaining 18% over 750 °C originates from Co_3_O_4_. Typical CV was carried out at a slow scan rate of 0.1 mV s^−1^ to analyze the electrochemical behaviors of electrodes (**Figure** **7**a). The cathodic peaks occurring at 2.3 and 2.05 V are due to the formation of high‐order LiPSs (Li_2_S*_n_*, 4 ≤ *n* ≤ 8) and further reduction to short‐chain insoluble Li_2_S_2_/Li_2_S. In contrast, one peak at ≈2.3–2.4 V implies oxidation process of LiPSs returning to S.^[^ ^66^ ^]^ The peak positions of S@Co_3_O_4_‐NP/N‐rGO cathode are almost unchanged during the first three cycles, indicating the highly reversibility and stability of LiPSs oxidation/reduction process. Besides, the cell with S@Co_3_O_4_‐NP/N‐rGO displays the higher slopes (the peak current *I*_p_ vs *v* ^0.5^) for all redox peaks and smaller polarizations than S@Co_3_O_4_‐NC/N‐rGO in Figure S20, Supporting Information, which proves its more favorable Li^+^ diffusion process. The charge–discharge profiles of the sulfur cathodes at different cycles under 0.2 C exhibit a charging slope and two distinctive discharge plateaus, which are consistent with the CV plots based on multistep polysulfide conversion reactions (Figure 7b). In addition, the S@Co_3_O_4_‐NP/N‐rGO electrode shows 337 mAh g^−1^ at the high discharge plateau and an initial discharge capacity of 1087 mAh g^−1^. As shown in Figure 7c, the S@Co_3_O_4_‐NP/N‐rGO electrode delivers a higher capacity retention of 84.1% than that of S@Co_3_O_4_‐NC/N‐rGO electrode (67.4%) after 100 cycles.

The rate performance of the S@Co_3_O_4_‐NP/N‐rGO electrode at various current densities is illustrated in Figure 7d,e. Clearly, the S@Co_3_O_4_‐NP/N‐rGO cathode shows discharge capacities of 1050, 915, 824, 702, and 569 mAh g^−1^ at the current rates of 0.2, 0.5, 1, 2, and 3 C, respectively. Even at the high current density of 3 C, the charge and discharge curves of the S@Co_3_O_4_‐NP/N‐rGO electrode still exhibit two discharge platforms, confirming its limited electrochemical overpotential and excellent reaction kinetics. The S@Co_3_O_4_‐NP/N‐rGO reveals the lower charge‐transfer resistance in Nyquist plots (Figure 7f), indicating the enhanced ion/electron transfer attributed to the atomic arrangements and surface electronic structures of Co_3_O_4_‐NP (112). In addition, the S@Co_3_O_4_‐NP/N‐rGO electrode shows a high capacity of 856 mAh g^−1^ at 1 C with a slow capacity fading rate (0.058% per cycle) whereas the S@Co_3_O_4_‐NC/N‐rGO cathode suffers much faster capacity fading rate (0.099% per cycle), which implies that the S@Co_3_O_4_‐NP/N‐rGO electrode has better sulfur utilization as well as reversible and stable reaction. Compared with S@Co_3_O_4_‐NC/N‐rGO cathode (91.4%), the average Coulombic efficiency of the S@Co_3_O_4_‐NP/N‐rGO electrode maintains at ≈98.8% during long‐term cell cycling (Figure 7g). The performance of electrode under raised sulfur loading was further assessed. In Figure 7h, the S@Co_3_O_4_‐NP/N‐rGO cathodes deliver a high areal capacity of 3.2 and 4.1 mAh cm^−2^ at an electrolyte/sulfur ratio of 8.8 and 5.3 mL g^−1^, respectively. These results fully demonstrate that S@Co_3_O_4_‐NP/N‐rGO electrode can effectively catalyze sulfur redox reactions, which offers significantly improved sulfur utilization and restrained LiPSs shuttle effect.

**Figure 8:**
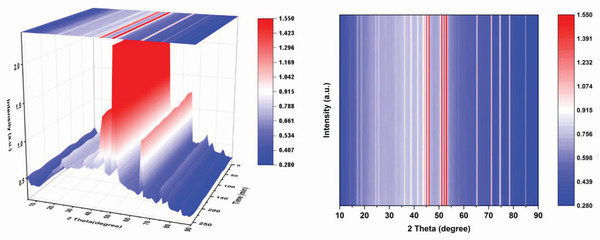
The operando X‐ray diffraction patterns of S@Co_3_O_4_‐NP/N‐rGO electrode.

In situ XRD studies of S@Co_3_O_4_‐NP/N‐rGO electrode were performed to investigate and determine structural changes during cycling. As shown in **Figure** **8** and Figure S21, Supporting Information, the characteristic patterns of Co_3_O_4_ can be clearly observed during the entire discharge/charge process, and its lattice parameters almost remain unchanged, while there is no featured peak of CoO phase (PDF No. 43‐1004), which indicates the high stability of Co_3_O_4_ as catalytic materials for Li–S battery cathodes.

## 3 Conclusion

In summary, we developed the Co_3_O_4_/N‐rGO composite via crystal surface engineering, which can be employed as an effective sulfur electrocatalyst to inhibit shuttle effect and accelerate the catalytic conversion of LiPSs. Co_3_O_4_ crystals enclosed by different planes with various Co^2+^_Td_/Co^3+^_Oh_ catalytic active sites are prepared to regulate the oxidation states of cobalt, the surface electronic structures, and the adsorption and catalytic capability. Compared with Co_3_O_4_‐NC crystals enclosed by (001) surfaces, the (112) lattice planes oriented Co_3_O_4_‐NP crystals with ample Co^3+^_Oh_ active sites strengthen the LiPSs affinity of Co_3_O_4_ and further enhance the LiPSs catalytic conversions. Meanwhile, the nitrogen‐doped rGO can effectively improve the electronic conductivity and electrolyte accessibility. Moreover, the Co_3_O_4_/N‐rGO composite architecture serves as a conductive framework to provide rich active interfaces and accelerate electron/ion transportation, rendering enhanced sulfur utilization, improved electrocatalytic behavior, and facilitated polysulfide conversion. Attributed to the structure superiorities, the S@Co_3_O_4_‐NP/N‐rGO electrode shows high specific capacity of 1087 mAh g^−1^ at 0.2 C, good rate performance, excellent cycling stability at 1 C corresponding to an exceptionally low capacity fading rate of 0.058%, and high areal capacity under raised sulfur loading. This work provides a perspective for synthesis of highly adsorptive and catalytic sulfur host materials and the development of practical Li–S batteries.

## Conflict of Interest

The authors declare no conflict of interest.

## Supporting information

Supporting Information

## Data Availability

The data that support the findings of this study are available from the corresponding author upon reasonable request.
