# Supplementary material for: Oxidation States Regulation of Cobalt Active Sites through Crystal Surface Engineering for Enhanced Polysulfide Conversion in Lithium–Sulfur Batteries
Source: Adv Sci (Weinh). 2022 Sep 15;9(31):2202352. doi: 10.1002/advs.202202352 (PMC9631056; doi:10.1002/advs.202202352)
Supplement: Supplementary file 1 — Supporting Information [file ADVS-9-2202352-s001.pdf]

ADVANCED SCIENCE
Open Access

Supporting Information

for *Adv. Sci.*, DOI 10.1002/advs.202202352

Oxidation States Regulation of Cobalt Active Sites through Crystal Surface Engineering for Enhanced Polysulfide Conversion in Lithium–Sulfur Batteries

Rujian Xiao, Dan Luo, Jiayi Wang, Han Lu, Heng Ma, Eser Metin Akinoglu, Mingliang Jin, Xin Wang*, Yongguang Zhang* and Zhongwei Chen**

Supporting Information

Oxidation States Regulation of Cobalt Active Sites through Crystal Surface Engineering for Enhanced Polysulfide Conversion in Lithium–Sulfur Batteries

Rujian Xiao, Dan Luo, Jiayi Wang, Han Lu, Heng Ma, Eser Metin Akinoglu, Mingliang Jin, Xin Wang,* Yongguang Zhang,* and Zhongwei Chen**

R. Xiao, Dr. D. Luo, J. Wang, Prof. M. Jin, Prof. X. Wang
South China Academy of Advanced Optoelectronics, School of Information and Optoelectronic Science and Engineering, South China Normal University, Guangdong 510006, China
E-mail: jinml@scnu.edu.cn; wangxin@scnu.edu.cn

H. Lu, Dr. E. M. Akinoglu, Prof. M. Jin, Prof. X. Wang, Prof. Y. Zhang
International Academy of Optoelectronics at Zhaoqing, South China Normal University, Zhaoqing 526060, China

H. Ma, Prof. Y. Zhang
School of Materials Science and Engineering, Hebei University of Technology, Tianjin 300130, China
E-mail: yongguangzhang@hebut.edu.cn

Dr. D. Luo, Prof. Z. Chen
Department of Chemical Engineering, University of Waterloo, Waterloo ON N2L 3G1, Canada
E-mail: zhwchen@uwaterloo.ca

Experimental Section

Synthesis of Co_3O_4-NC/N-rGO: Co_3O_4 nanocrystals loaded on nitridation-reduced graphene with different shapes were prepared by one-step hydrothermal method. For Co_3O_4-NC/N-rGO composites, graphene aqueous dispersion (22 ml, 1 mg mL^{-1}) was prepared and ultrasonized for half hour, followed by $Co(NO_3)_2$ aqueous solution (1.0 mL, 1 mol L^{-1}), $NaNO_3$ (0.0425 g) and $NH_3 \cdot H_2O$ (2.0 mL, 25 wt%). The solution was stirred at room temperature for 4 hours, then transferred to a Teflon autoclave heating at 185 °C for 12 hours. The products were obtained by centrifugation after washing with distilled water and ethanol, subsequently dried at 60 °C for 12 h.

Synthesis of Co_3O_4-NP/N-rGO: For the preparation of Co_3O_4-NP/N-rGO, the experimental procedures follow the similar conditions to the of Co_3O_4-NC/N-rGO except with slight variations. Typically, GO aqueous solution (16 mL, 1 mg mL^{-1}), $Co(NO_3)_2$ (1.0 mL, 1 mol L^{-1}), $NaNO_3$ (0.0425 g), and $NH_3 \cdot H_2O$ (8.0 mL, 25 wt%) were mixed together and other steps remain the same.

Synthesis of S@Co_3O_4/N-rGO: A typical melt-diffusion method is used to load sulfur. The sulfur was mixed and milled with as-prepared Co_3O_4/N-rGO at a mass ratio of 3:1, then sealed in a clean Teflon pot and kept at 155 °C for 12 h.

Preparation of Li_2S_6 Solution: Sulfur and Li_2S were dissolved in the electrolyte with a molar ratio of 5:1, and then stirred in Ar-filled glove box at 55 °C for 24 h to obtain a concentration of 0.1 M Li_2S_6 electrolyte. In addition, Li_2S_6/THF solution was prepared via the same method for the polysulfide UV–vis spectroscopy test.

Materials Characterization: The crystal structures and phases of the samples were determined by a Bruker D8 Advance X-ray diffractometer. Raman spectra were obtained on a Renishaw inVia Raman spectrometer system. The micro-nanostructures of all materials were investigated by scanning electron microscopy (SEM, ZEISS Ultra 55) and transmission electron microscopy (TEM, JEOL, JEM-2100), respectively. To characterize the elemental valence states of Co_3O_4/N-rGO, X-ray photoelectron spectroscopy (XPS, Thermo, ESCALAB 250) was performed. According to the Brunauer–Emmett–Teller (BET) equation, nitrogen adsorption–desorption measurements (Mikete, ASAP2020) were conducted to

calculate the specific surface area. Thermal gravimetric analysis (TGA, LABSYS EVO) was carried out at a ramping heating rate of 10 °C min^{-1} under N_2 atmosphere in a temperature range from 25 to 900 °C.

Electrochemical Characterization: The sulfur electrodes were prepared by mixing carbon black, polyvinylidene fluoride (PVDF) and S@Co_3O_4/N-rGO composites at a mass ratio of 1:1:8 in NMP dispersant solvent. Then the slurry was compressed onto aluminum foil and dried at 60 °C for 12 h. The regular mass loading of sulfur for each electrode about 1 mg cm^{-2}. CR2032 type coin cells were assembled with S@Co_3O_4/N-rGO composite cathode, lithium foil anode, polypropylene separator (Celgard) and 1.0 M LiTFSI in DOL:DME=1:1 Vol% (0.1 M $LiNO_3$) electrolyte in an argon-filled glove-box. And an amount of 30 μL electrolyte was added in each cell. Cycle performance were examined by Neware (BST-5 V 5 mA) tester under galvanostatic discharge–charge with cutoff voltages of 1.7–2.8 V. An electrochemical station was employed to conduct the cyclic voltammetry (CV) and electrochemical impedance spectroscopy (EIS) measurements.

Symmetric Cells Assembly and Measurement: Two identical Co_3O_4/N-rGO electrodes with a mass loading of 1 mg cm^{-2} acted as the working and the counter electrodes and 40 uL Li_2S_6 electrolyte (0.1 M) was used as cell electrolyte. The CV curves were examined at a scan rate of 10 mV s^{-1} within a voltage window between −1.5 and 1.5 V. EIS spectra were also collected in a frequency range of 0.01 Hz to 100 kHz. The ion diffusivity was measured based on the equation:

$$I_p = 269000 \times n^{1.5} \times A \times C \times D^{0.5} \times v^{0.5} \quad (1)$$

in which I_p refers to the peak current, n means the number of electrons, A is the electrode area, C represents the Li^+ concentration, D is Li^+ diffusion coefficient and *v* corresponds to the scan rate.[1,2]

Linear Sweep Voltammetry Test: The LSV measurements were caried out using Co_3O_4/N-rGO composites (working electrode), saturated Ag/AgCl electrode (reference electrode), platinum sheet (counter electrode) and 0.1 M Li_2S/methanol solution at a scan rate of 10 mV s^{-1} from −0.8 to −0.1 V. For the working electrode preparation, the slurry containing PVDF binder, carbon black, Co_3O_4/N-rGO composites at a mass ratio of 1:1:8 was dropped onto bare glass carbon.

Nucleation of Lithium Sulfide Test: Co_3O_4/N-rGO materials loaded on carbon paper, lithium foil, 0.1M Li_2S_8/tetraglyme solution were used as cathode, anode, and electrolyte to assemble coin cells, respectively. The batteries were first galvanostatically discharged at a current of 0.112 mA to 2.06 V, then potentiostatic discharging at 2.05 V until the current dropped to 0.01 mA.

Computational Details: The first-principles were employed to perform spin-polarization density functional theory (DFT) calculations with the projector augmented wave (PAW) method. The exchange-correlation energies were calculated using Perdew−Burke−Ernzerhof generalized−gradient approximation (GGA) functions. Besides, the ionic cores interactions and valence electrons were described in a plane-wave basis set with an energy cutoff of 400 eV and the vacuum spacing is 15 Å. The electronic energy was self-consistent and geometries converged until the energy was less than 10^{-6} eV and 0.05 eV/Å, respectively. Moreover, the U schema is used for Co atom and the U value is set to 3.2 eV. The Brillouin zone integration is performed with a Gaussian smearing of 0.05 eV over a 3×3×1 Monkhorst−Pack k-point sampling for partial occupancies of Kohn−Sham orbitals. To explore the interactions, the adsorption energies (E_{ads}) of Li_2S_6 on Co_3O_4 (001) and Co_3O_4 (112) surfaces were calculated. The adsorption energies (E_{ads}) are defined as: $E_{ads}= E_{ad/sub}-E_{ad}-E_{sub}$, where, $E_{ad/sub}$ is the total energy of the system containing Co_3O_4 surface with adsorbed LiPSs, E_{ad} is the adsorbate in the structure, and E_{sub} is the clean substrate, respectively.

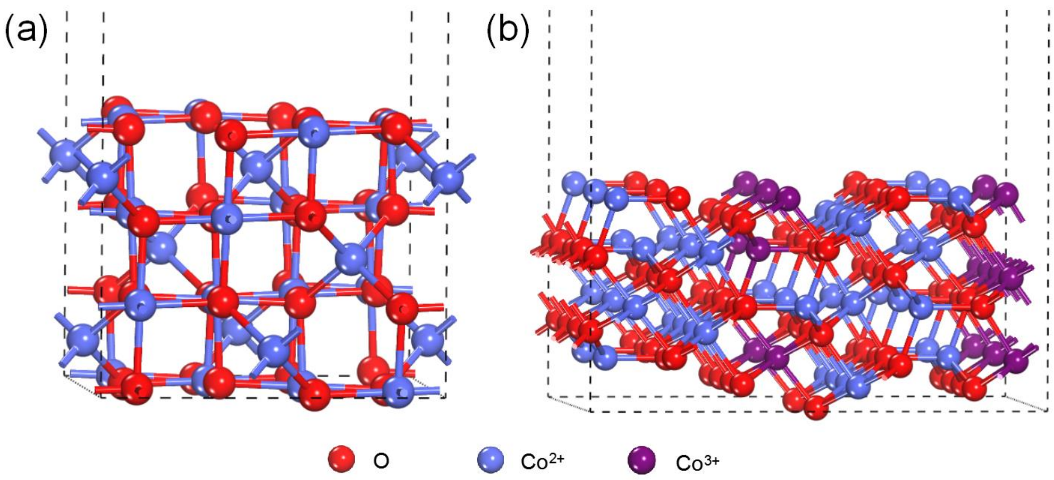

Figure S1. The Co^{2+}/Co^{3+} surface atomic configurations on (a) Co_3O_4 (001) and (b) Co_3O_4 (112) planes.

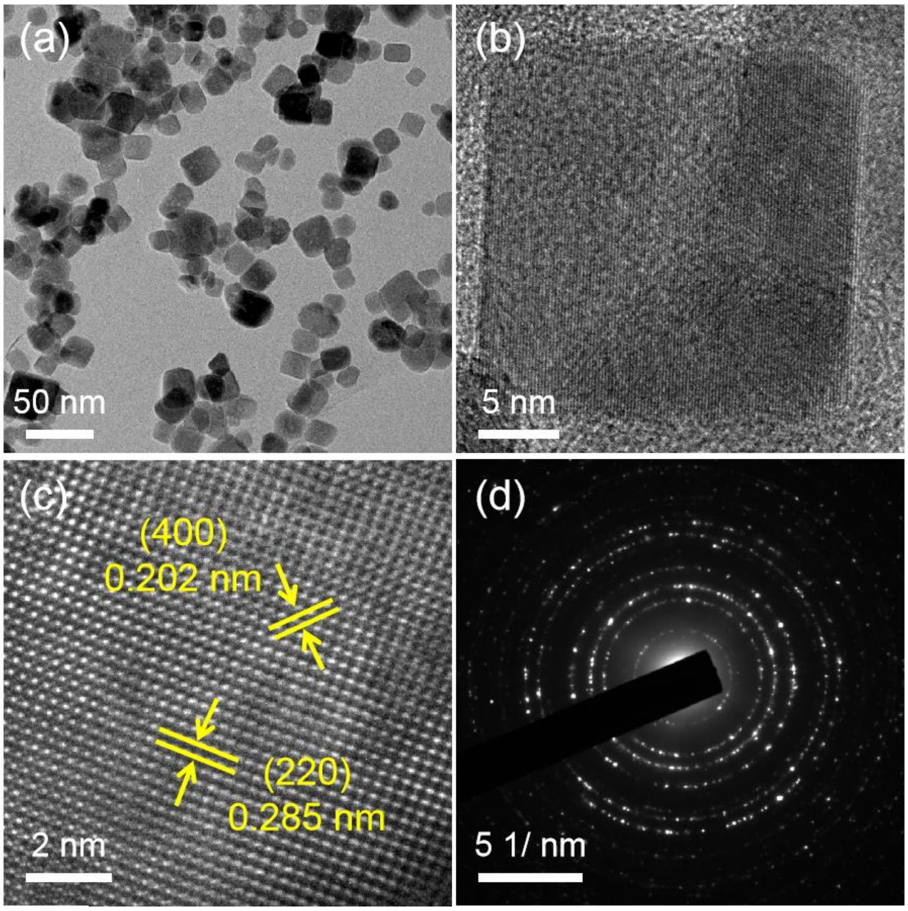

Figure S2. (a) TEM, (b, c) HRTEM images and (d) SAED pattern of Co_3O_4-NC/N-rGO.

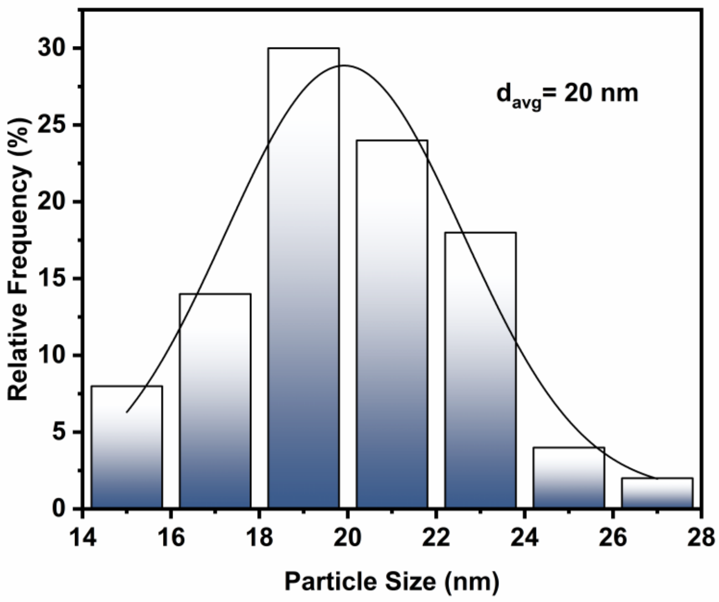

Figure S3. Particle size distribution of Co_3O_4-NC crystals on N-rGO.

.

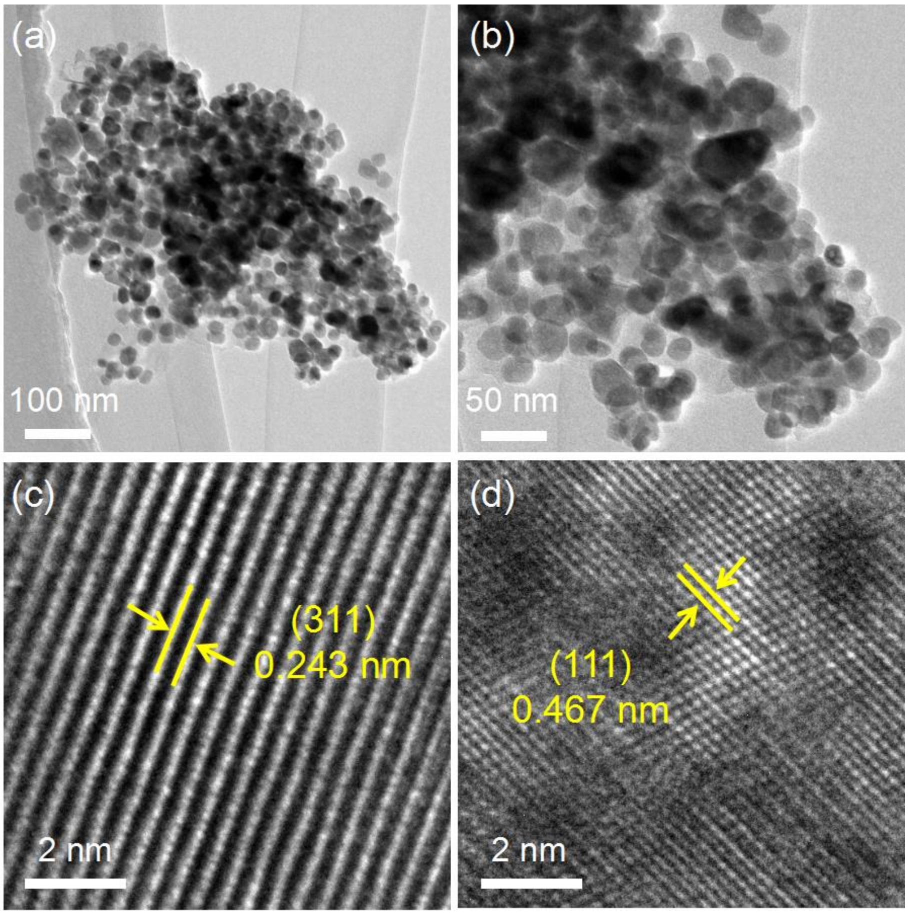

Figure S4. (a, b) TEM images and (c, d) HRTEM images of Co_3O_4-NP/N-rGO.

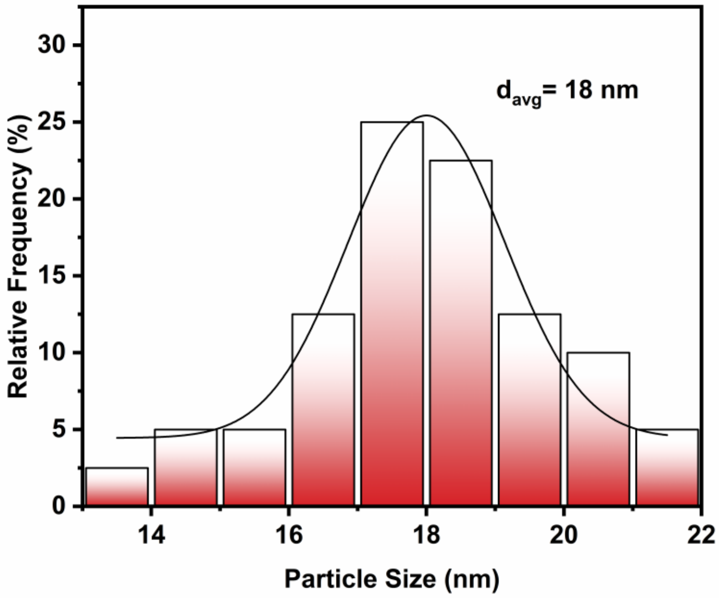

Figure S5. Particle size distribution of Co_3O_4-NP crystals on N-rGO.

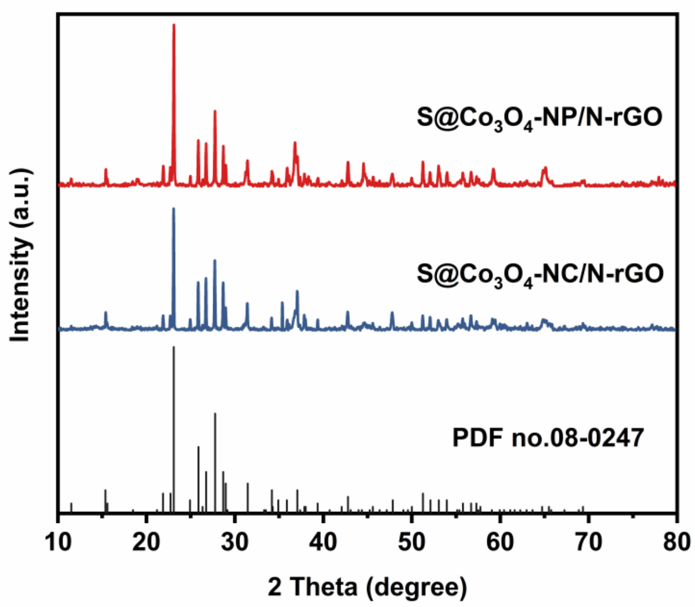

Figure S6. XRD patterns of S@Co_3O_4-NC/N-rGO and S@Co_3O_4-NP/N-rGO.

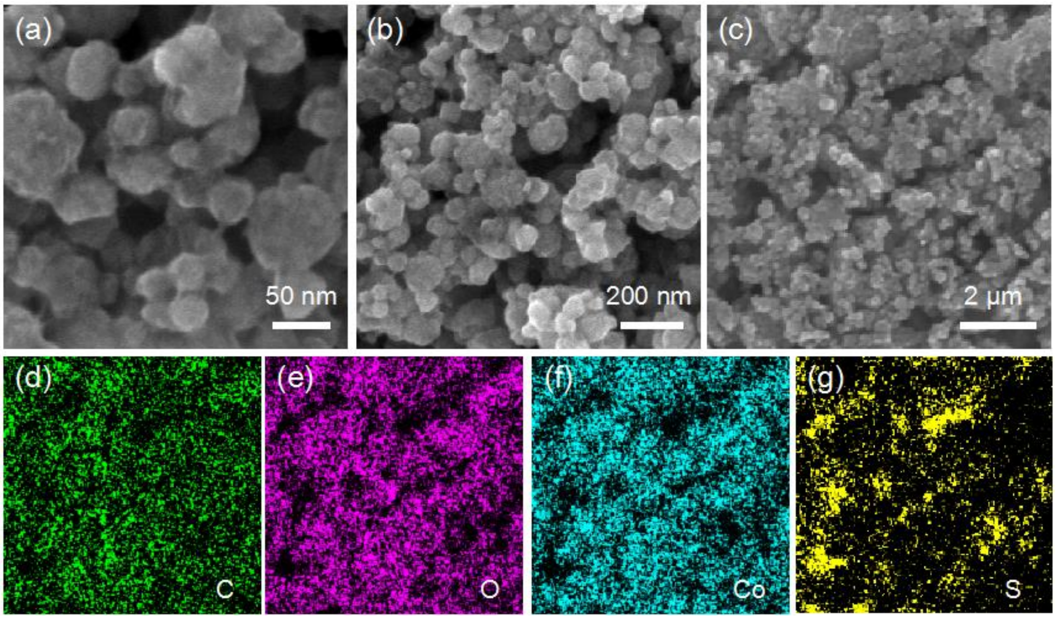

Figure S7. (a–c) SEM images of S@Co_3O_4-NP/N-rGO. (d–g) Elemental mapping of S@Co_3O_4-NP/N-rGO.

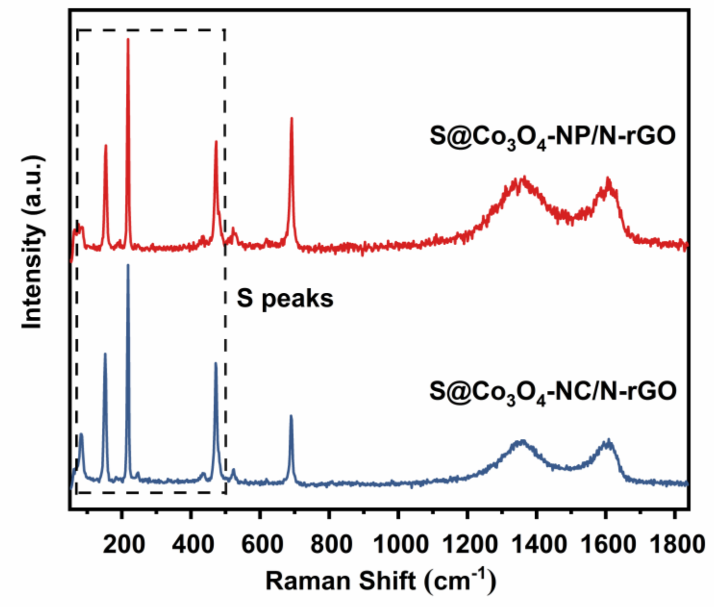

Figure S8. Raman spectra of S@Co_3O_4-NC/N-rGO and S@Co_3O_4-NP/N-rGO.

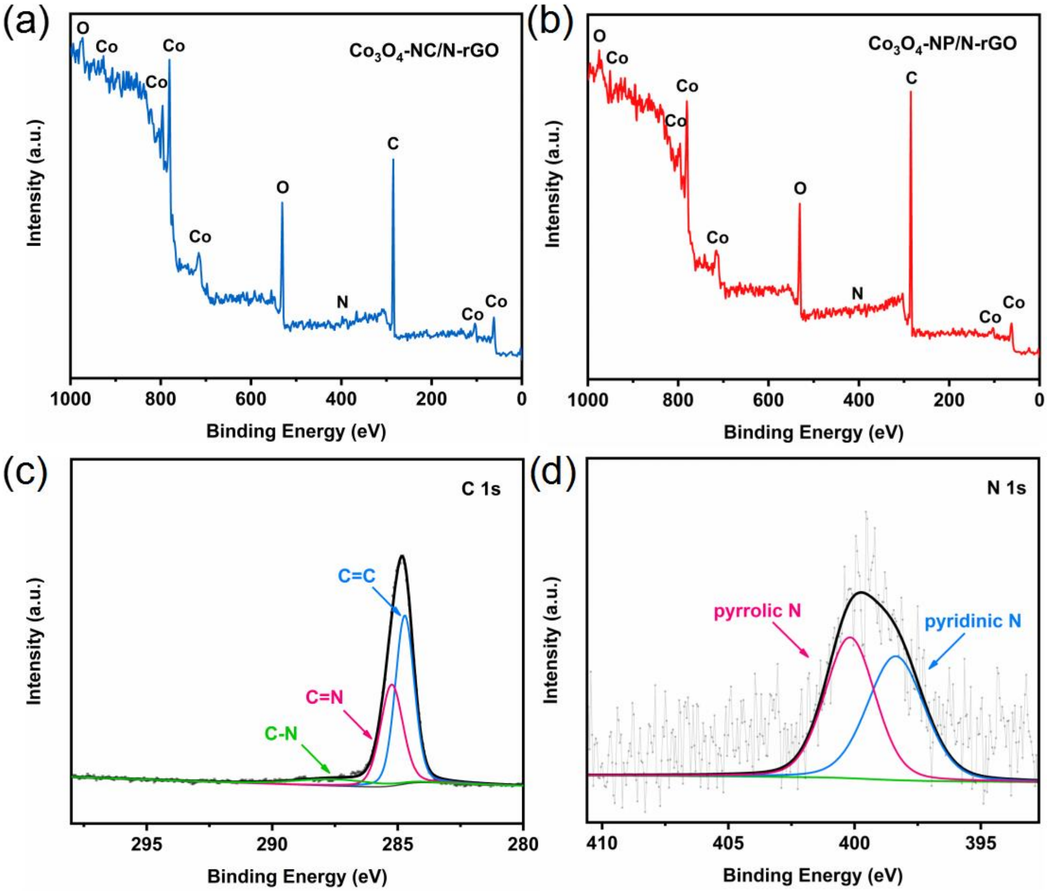

Figure S9. XPS survey spectra of (a) Co_3O_4-NC/N-rGO and (b) Co_3O_4-NP/N-rGO. (c) C 1s and (d) N 1s core level in Co_3O_4-NC/N-rGO.

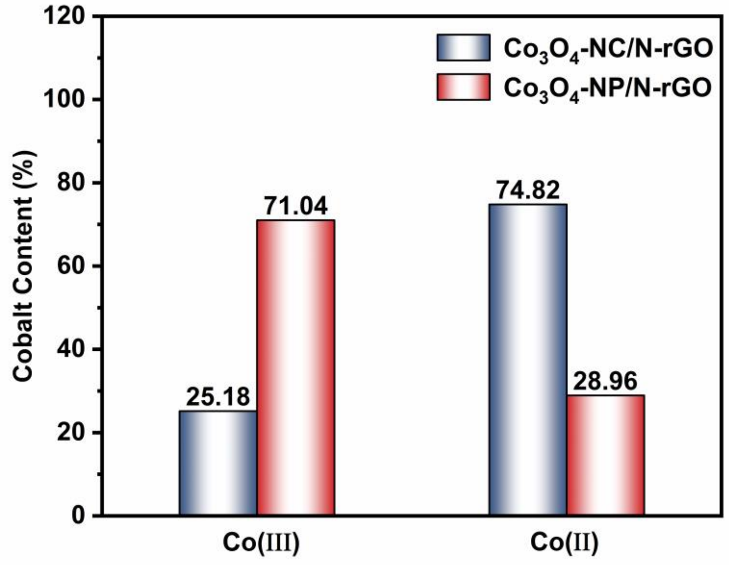

Figure S10. Cobalt content in the Co_3O_4-NP/N-rGO and Co_3O_4-NC/N-rGO.

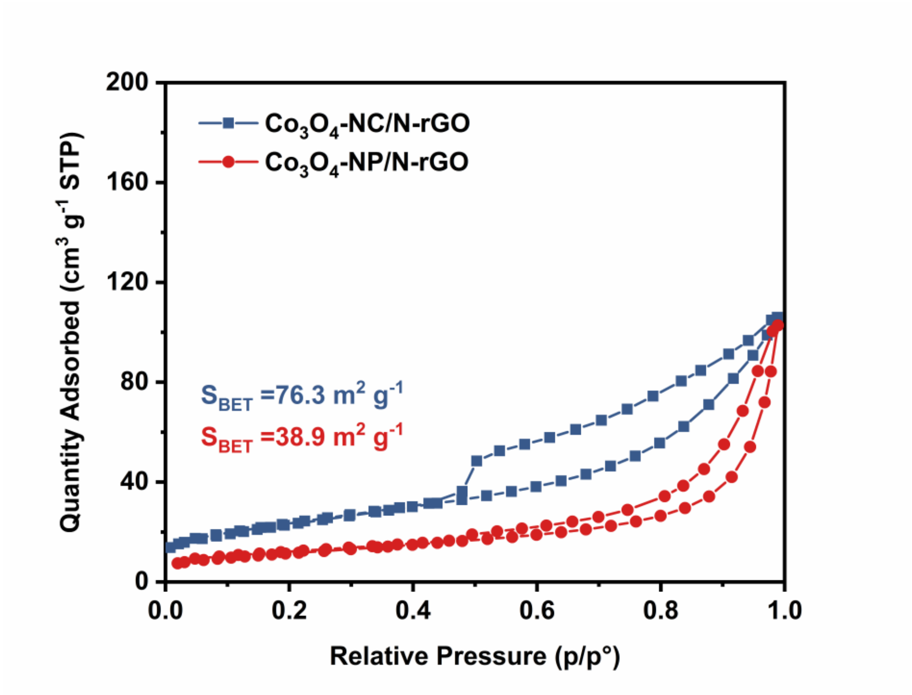

Figure S11. N_2 adsorption–desorption isotherm curves of Co_3O_4-NC/N-rGO and Co_3O_4-NP/N-rGO.

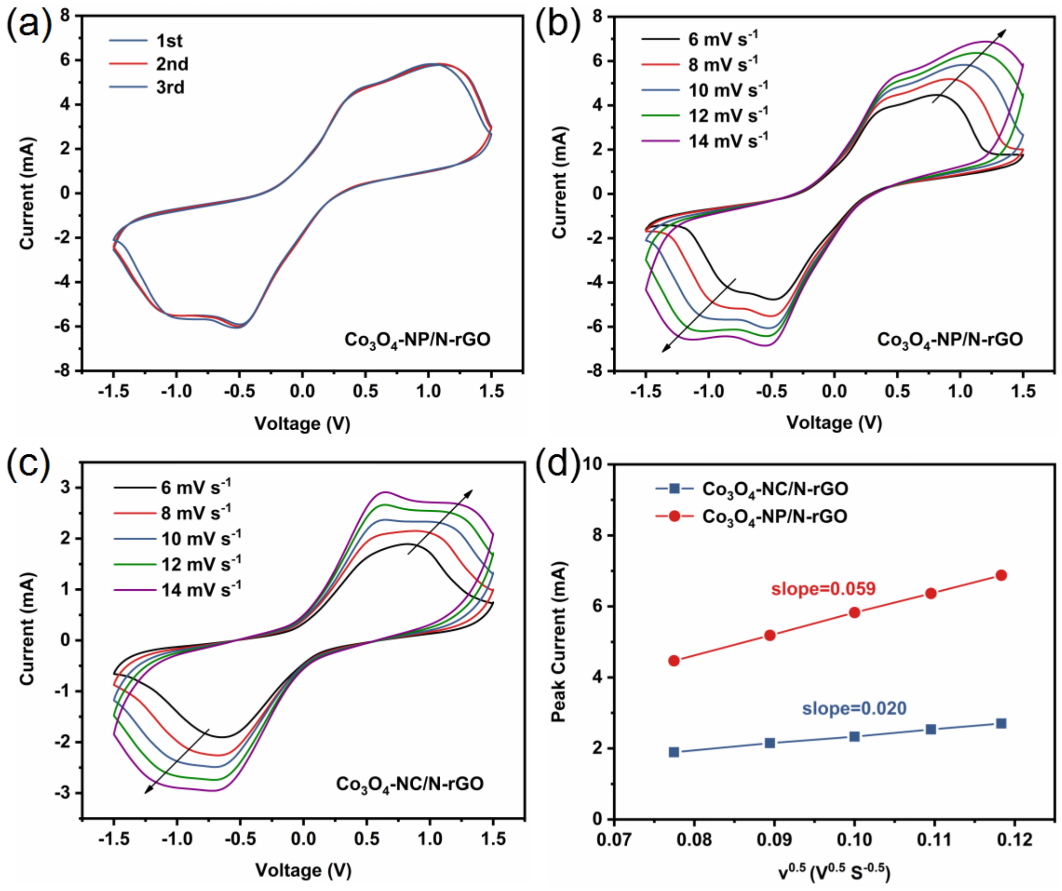

Figure S12. (a–c) CV curves of Co_3O_4-NP/N-rGO and Co_3O_4-NC/N-rGO symmetric cells. (d) The corresponding linear fitting based on Randlesv–Sevick equation.

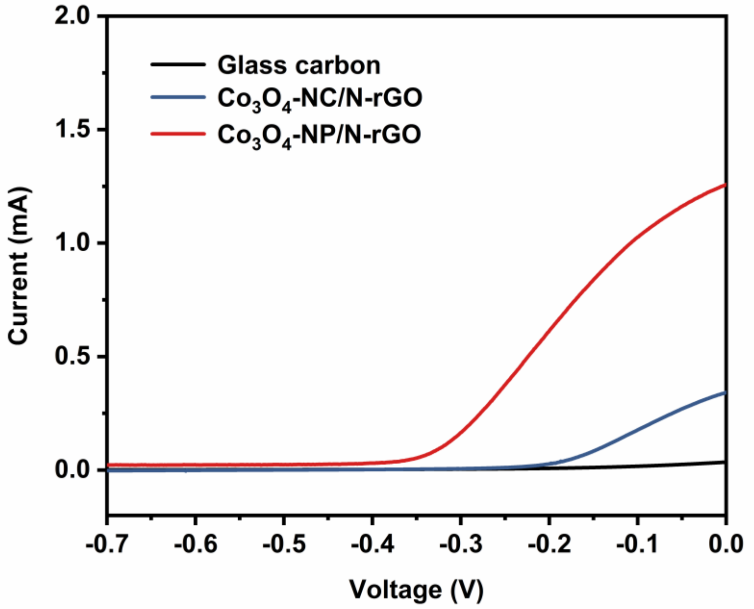

Figure S13. LSV curves of Li_2S oxidization on different Co_3O_4/N-rGO surfaces.

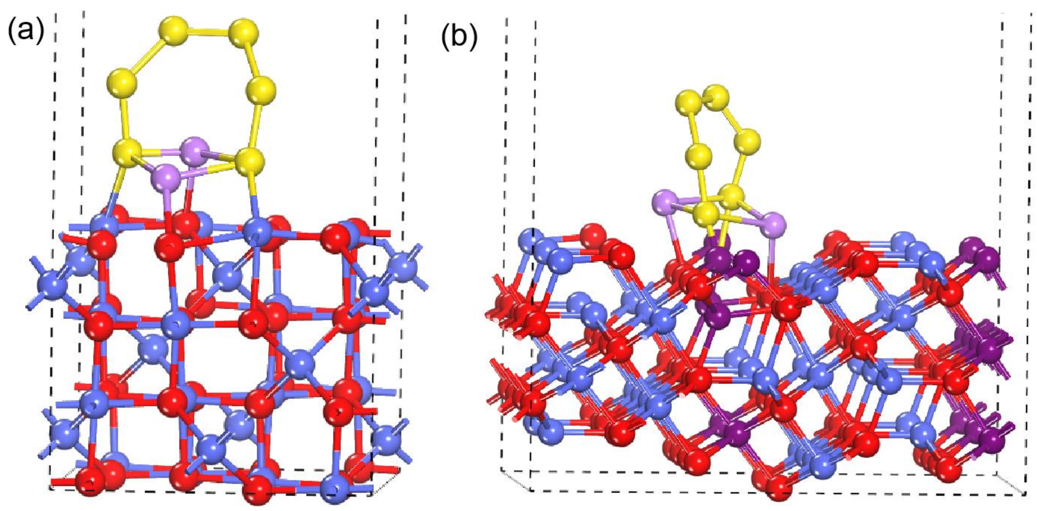

Figure S14. Geometrically stable configurations of Li_2S_6 adsorption on (a) Co_3O_4 (001) and (b) Co_3O_4 (112) surfaces.

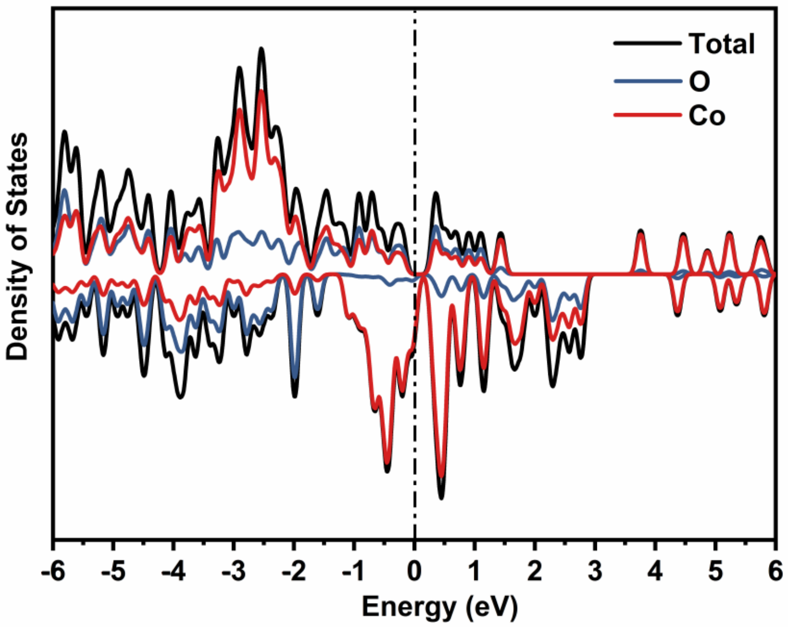

Figure S15. Total and partial DOS of Co_3O_4 (001).

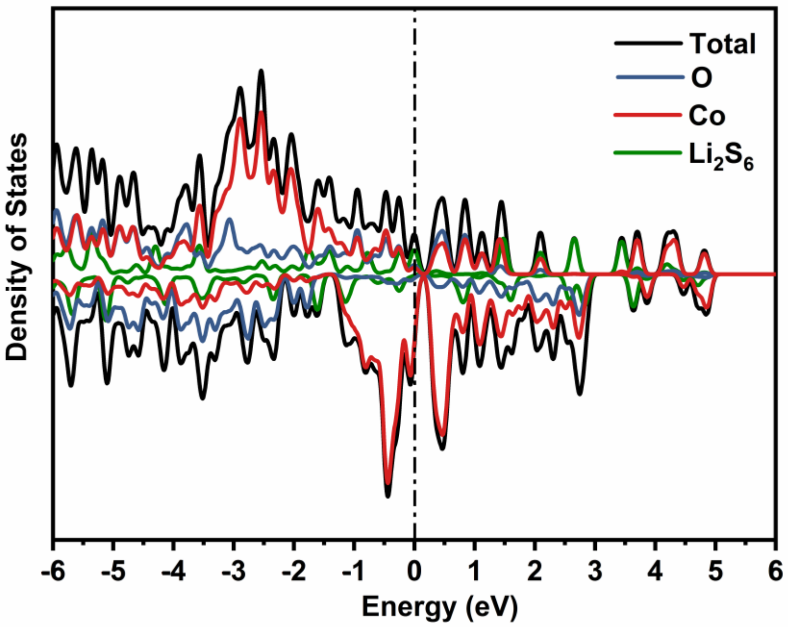

Figure S16. Total and partial DOS of Li_2S_6-Co_3O_4 (001)-Co^{2+}.

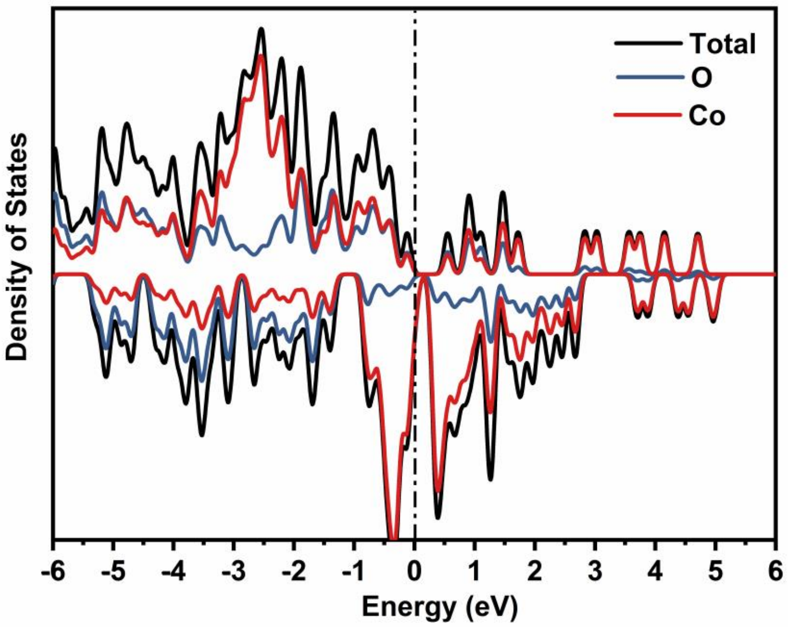

Figure S17. Total and partial DOS of Co_3O_4 (112).

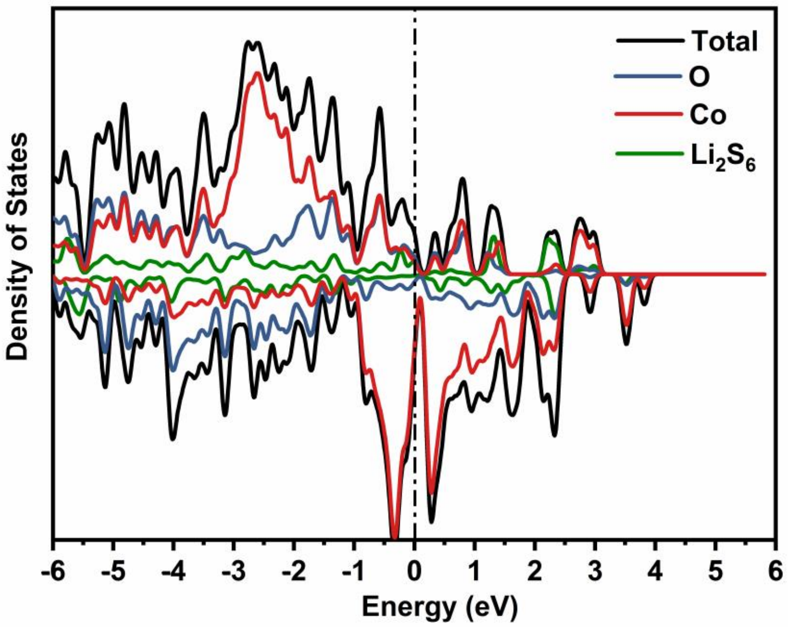

Figure S18. Total and partial DOS of Li_2S_6-Co_3O_4 (112)-Co^{3+}.

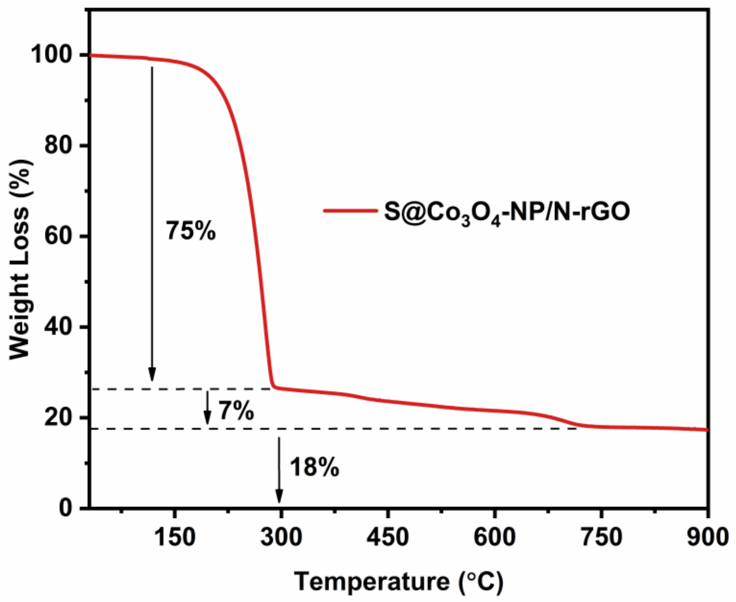

Figure S19. TGA curves of S@Co_3O_4-NP/N-rGO composites.

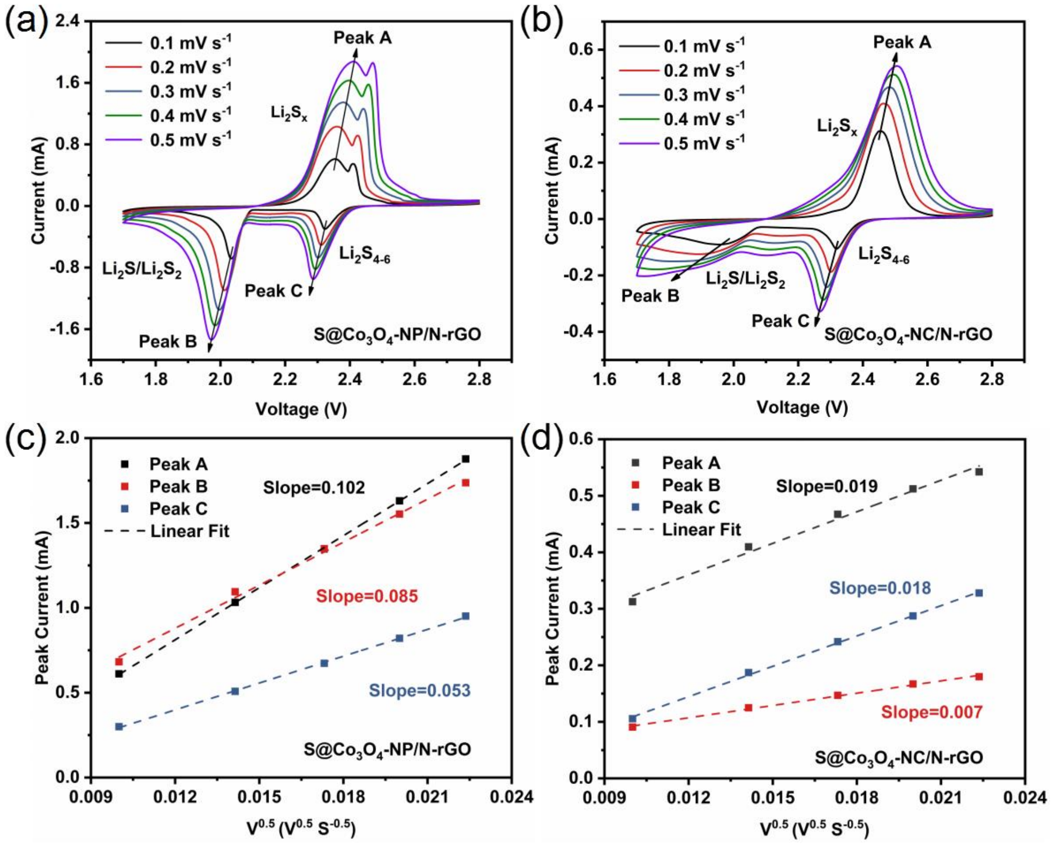

Figure S20. CV curves at varied scanning rates and the corresponding linear fittings between I_p and $v^{0.5}$ for (a, c) S@Co_3O_4-NP/N-rGO, and (b, d) S@Co_3O_4-NC/N-rGO electrodes.

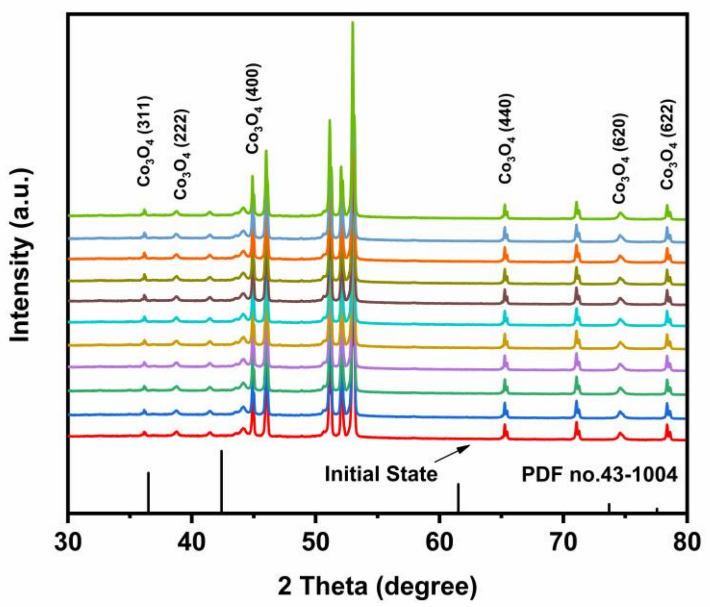

Figure S21. The operando XRD patterns of S@Co_3O_4-NP/N-rGO electrode.

Table S1. Elemental compositions of prepared two Co_3O_4/N-rGO composites.

Sample	O（at%）	N（at%）	C（at%）	Co（at%）
Co_3O_4-NC/N-rGO	23.05	1.43	66.33	9.19
Co_3O_4-NP/N-rGO	16.42	1.62	76.30	5.66

References

[1] K. Wu, Y. Hu, Z. Shen, R. Chen, X. He, Z. Cheng, P. Pan, *J. Mater. Chem. A* **2018**, *6*, 2693-2699.

[2] Z. A. Ghazi, X. He, A. M. Khattak, N. A. Khan, B. Liang, A. Iqbal, J. Wang, H. Sin, L. Li, Z. Tang, *Adv. Mater.* **2017**, *29*, 1606817-1606823.
